# Carbolic Acid in Treatment of Dysentery

**Published:** 1873-07

**Authors:** 


### CARBOLIC ACID IN TREATMENT OF DYSENTERY.

Dr. Amelung, of Carlshafen, Germany, has used carbolic acid in an epidemic of dysentery with unusual success. Out of eighty patients he lost only two—one elderly lady with peritonitis following the primary disease, and one rather delicate child six months old.

The fact that this agent has in the last few years been extensively and successfully used for the disinfection of wounds and putrid secretions, in pharyngeal diphtheritis, locally for the destruction of the exudation, in gangrene of the lungs, in tedious intestinal catarrhs of children, and in zymotic diseases generally, induced him to test its use in this disease, which, when it occurs epidemically is, according to pathological anatomy, diphtheritis of the large intestines. After removing hard fecal substances by an emulsion of castor oil, he uses about one grain doses of the carbolic acid* every two hours, in grown persons—less of course in children, according to their age—so that about fifteen grains are consumed in course of two days. In the majority of cases the dysenteric discharge and the tenesmus disappear, and thin, feculent stools set in, under this treatment, in course of two, seldom four to five days. This change is considered a favorable prognostic sign. The thin discharges generally yield to tannin with opium, and when inclined to be tedious, to tincture catechu. A close attention to the disinfection of the vessels containing the discharges, and also of the privies by crude carbolic acid, is considered of great importance.—*Berliner Klinische Wochenschrift, No.* 11, 1873. [The large number of cases which Dr. Amelung had to attend within a very short period of time did not permit him to test the local effect of carbolic acid directly applied to the diseased mucous membrane, in the form of enemata. R.

* The following is the formula usually prescribed by him, the quantities stated in grammes, one gramme being equal to fifteen grains: R.—Acid carbol 1.9, aqua distil 150.0, mucilag. gum arabic, syrup aa 25.0. Sig.—One tablespoonful every two hours.

